# [*N*-(5-Bromo-2-oxidobenzyl­idene)-l-valin­ato-κ^3^ *O*,*N*,*O*′]diethyl­tin(IV)

**DOI:** 10.1107/S1600536808032388

**Published:** 2008-10-15

**Authors:** Wei Wang, Laijin Tian, Dengtai Chen, Yuan Qu

**Affiliations:** a Department of Chemistry, Qufu Normal University, Qufu 273165, People’s Republic of China; b Department of Chemistry, Shandong University, Jinan 250100, People’s Republic of China

## Abstract

The Sn atom of the title compound, [Sn(C_2_H_5_)_2_(C_12_H_12_BrNO_3_)], is in a distorted SnNC_2_O_2_ trigonal–bipyramidal geometry and forms five- and six-membered chelate rings with the tridentate ligand. One C atom of one ethyl group is disordered with site occupancies of 0.61 (3):0.39 (3).

## Related literature

For related structures, see: Beltran *et al.* (2003 ▶); Basu Baul *et al.* (2007 ▶); Dakternieks *et al.* (1998 ▶); Rivera *et al.* (2006 ▶); Tian *et al.* (2005 ▶, 2006 ▶, 2007 ▶).
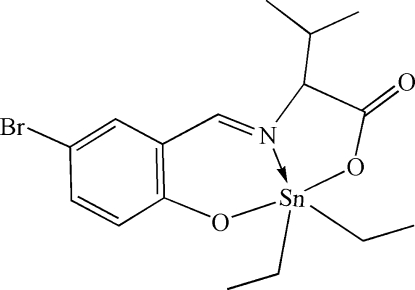


## Experimental

### 

#### Crystal data

[Sn(C_2_H_5_)_2_(C_12_H_12_BrNO_3_)]
*M* *_r_* = 474.95
Orthorhombic, 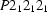

*a* = 9.810 (2) Å
*b* = 10.377 (2) Å
*c* = 18.301 (4) Å
*V* = 1863.1 (7) Å^3^
*Z* = 4
Mo *K*α radiation
μ = 3.53 mm^−1^
*T* = 295 (2) K
0.20 × 0.18 × 0.11 mm

#### Data collection

Bruker SMART APEX area-detector diffractometer
Absorption correction: multi-scan (*SADABS*; Bruker, 2002 ▶) *T*_min_ = 0.519, *T*_max_ = 0.688
15137 measured reflections
3836 independent reflections
3292 reflections with *I* > 2σ(*I*)
*R*_int_ = 0.038

#### Refinement

*R*[*F* ^2^ > 2σ(*F* ^2^)] = 0.035
*wR*(*F* ^2^) = 0.085
*S* = 1.04
3836 reflections
209 parameters
H-atom parameters constrained
Δρ_max_ = 0.34 e Å^−3^
Δρ_min_ = −0.85 e Å^−3^
Absolute structure: Flack (1983 ▶), 1634 Friedel pairs
Flack parameter: 0.017 (14)

### 

Data collection: *SMART* (Bruker, 2002 ▶); cell refinement: *SAINT* (Bruker, 2002 ▶); data reduction: *SAINT*; program(s) used to solve structure: *SHELXS97* (Sheldrick, 2008 ▶); program(s) used to refine structure: *SHELXL97* (Sheldrick, 2008 ▶); molecular graphics: *ORTEP-3 for Windows* (Farrugia, 1997 ▶); software used to prepare material for publication: *SHELXL97*.

## Supplementary Material

Crystal structure: contains datablocks global, I. DOI: 10.1107/S1600536808032388/tk2313sup1.cif

Structure factors: contains datablocks I. DOI: 10.1107/S1600536808032388/tk2313Isup2.hkl

Additional supplementary materials:  crystallographic information; 3D view; checkCIF report

## Figures and Tables

**Fig. 1.**
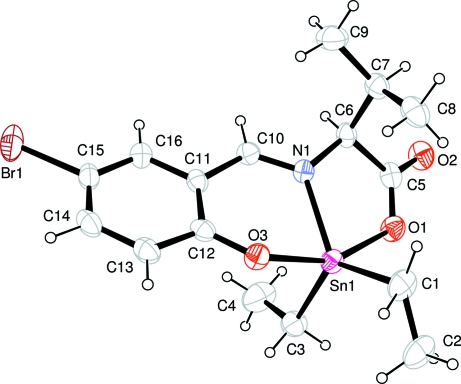
The molecular structure of (I) with displacement ellipsoids drawn at the 30% probability level. For C2 atom of ethyl group, the minor disordered component has been omitted for clarity.

## supplementary crystallographic information

### Comment

The structural chemistry of diorganotin complexes with Schiff bases derived from α-amino acids has received attention due to their biological activities and their nonlinear optical properties (Beltran *et al.*, 2003; Basu Baul *et al.*, 2007; Dakternieks *et al.*, 1998; Rivera *et al.*, 2006; Tian *et al.*, 2005, 2006, 2007). The structures of several diorganotin complexes with the Schiff base ligand [*N*-(2-oxidohydroxyphenylmethylene)valine, such as [*N*-(2-oxidophenylmethylene)valinato]dibutyltin(IV), [*N*-(2-oxidophenylmethylene)valinato]diphenyltin(IV) (Beltran *et al.*, 2003), [*N*-(4-diethylamino-2-oxidophenylmethylene)valinato]diphenyltin(IV) (Rivera *et al.*, 2006), [*N*-(5-bromo-2-oxidophenylmethylene)valinato]diphenyltin(IV), (Tian *et al.*, 2005) have been reported. As a continuation of these studies, the structure of the title compound, (I), is now described.

The coordination geometry about the tin atom in (I) is that of a distorted trigonal bipyramid with two ethyl groups and the imino N1 atom occupying the equatorial positions and the axial positions being occupied by a unidentate carboxylate O1 atom and phenoxide O3 atom (Fig. 1). The tin atom is 0.063 (2) Å out of the NC_2_ trigonal plane in the direction of the O3 atom. The bond length of Sn1—O1 (2.153 (3) Å) is longer than that of Sn1—O3 (2.106 (3) Å) and the O1—Sn1—O3 bond angle is 156.21 (13) °. The monodentate mode of coordination of the carboxylate is reflected in the disparate C5—O1 and C5—O2 bond lengths of 1.287 (6) and 1.221 (6) Å, respectively.

### Experimental

The title compound was synthesized by the reaction of diethyltin dichloride (0.50 g, 2 mmol) with potassium *N*-(5-bromosalicylidene)valinate (0.68 g, 2 mmol) in the presence of Et_3_N (0.20 g, 2 mmol) in  methanol (50 ml). The reaction mixture was refluxed for 3 h and filtered. The yellow solid (I) was obtained by removal of solvent under reduced pressure and was recrystallized from methanol. Crystals for crystallography were obtained from the slow evaporation of a chloroform-hexane (1:1, v/v) solution of (I) held at room temperature (yield 71%, m.p. 479–480 K).

### Refinement

The C2 atom of one ethyl group was disordered over two positions; the site occupancy was refined to 0.61 (3):0.39 (3). The absolute configuration of the compound (I) was assigned on the basis of the known configuration of the starting reagent, L-valine. H atoms were placed at calculated positions and were included in the refinement in the riding-model approximation, with C—H = 0.93 - 0.98 Å, and with *U*_iso_(H) = 1.2-1.5U_eq_(C).

### Crystal data

**Table:**

[Sn(C_2_H_5_)_2_(C_12_H_12_BrNO_3_)]	*F*(000) = 936
*M**_r_* = 474.95	*D*_x_ = 1.693 Mg m^−^^3^
Orthorhombic, *P*2_1_2_1_2_1_	Mo *K*α radiation, λ = 0.71073 Å
Hall symbol: P 2ac 2ab	Cell parameters from 4436 reflections
*a* = 9.810 (2) Å	θ = 2.2–22.1°
*b* = 10.377 (2) Å	µ = 3.53 mm^−^^1^
*c* = 18.301 (4) Å	*T* = 295 K
*V* = 1863.1 (7) Å^3^	Block, yellow
*Z* = 4	0.20 × 0.18 × 0.11 mm

### Data collection

**Table:**

Bruker SMART APEX area-detector diffractometer	3836 independent reflections
Radiation source: fine-focus sealed tube	3292 reflections with *I* > 2σ(*I*)
graphite	*R*_int_ = 0.038
φ and ω scans	θ_max_ = 26.5°, θ_min_ = 2.2°
Absorption correction: multi-scan (SADABS; Bruker, 2002)	*h* = −12→12
*T*_min_ = 0.519, *T*_max_ = 0.688	*k* = −12→13
15137 measured reflections	*l* = −22→22

### Refinement

**Table:**

Refinement on *F*^2^	Secondary atom site location: difference Fourier map
Least-squares matrix: full	Hydrogen site location: inferred from neighbouring sites
*R*[*F*^2^ > 2σ(*F*^2^)] = 0.035	H-atom parameters constrained
*wR*(*F*^2^) = 0.085	*w* = 1/[σ^2^(*F*_o_^2^) + (0.0368*P*)^2^ + 0.0992*P*] where *P* = (*F*_o_^2^ + 2*F*_c_^2^)/3
*S* = 1.04	(Δ/σ)_max_ = 0.001
3836 reflections	Δρ_max_ = 0.34 e Å^−^^3^
209 parameters	Δρ_min_ = −0.85 e Å^−^^3^
0 restraints	Absolute structure: Flack (1983), 1634 Friedel pairs
Primary atom site location: structure-invariant direct methods	Flack parameter: 0.017 (14)

### Special details

**Table:**

Geometry. All e.s.d.'s (except the e.s.d. in the dihedral angle between two l.s. planes) are estimated using the full covariance matrix. The cell e.s.d.'s are taken into account individually in the estimation of e.s.d.'s in distances, angles and torsion angles; correlations between e.s.d.'s in cell parameters are only used when they are defined by crystal symmetry. An approximate (isotropic) treatment of cell e.s.d.'s is used for estimating e.s.d.'s involving l.s. planes.
Refinement. Refinement of *F*^2^ against ALL reflections. The weighted *R*-factor *wR* and goodness of fit *S* are based on *F*^2^, conventional *R*-factors *R* are based on *F*, with *F* set to zero for negative *F*^2^. The threshold expression of *F*^2^ > σ(*F*^2^) is used only for calculating *R*-factors(gt) *etc*. and is not relevant to the choice of reflections for refinement. *R*-factors based on *F*^2^ are statistically about twice as large as those based on *F*, and *R*- factors based on ALL data will be even larger.

### Fractional atomic coordinates and isotropic or equivalent isotropic displacement parameters (Å^2^)

**Table:**

	*x*	*y*	*z*	*U*_iso_*/*U*_eq_	Occ. (<1)
Sn1	0.98675 (3)	0.99102 (3)	0.941187 (17)	0.05242 (11)	
N1	0.7827 (4)	1.0416 (3)	0.9737 (2)	0.0458 (9)	
O1	1.0064 (4)	1.1614 (3)	1.00747 (18)	0.0660 (8)	
O2	0.9042 (4)	1.3037 (3)	1.0809 (2)	0.0753 (11)	
O3	0.8849 (3)	0.8346 (3)	0.89231 (19)	0.0590 (8)	
Br1	0.35058 (8)	0.84038 (8)	0.73311 (4)	0.0989 (3)	
C1	1.1209 (6)	0.8765 (6)	1.0041 (4)	0.0768 (17)	
H1A	1.0733	0.8479	1.0476	0.092*	
H1B	1.1448	0.8005	0.9760	0.092*	
C2'	1.2499 (13)	0.9437 (18)	1.0268 (15)	0.100 (8)	0.61 (3)
H2D	1.3056	0.8855	1.0545	0.149*	0.61 (3)
H2E	1.2276	1.0173	1.0563	0.149*	0.61 (3)
H2F	1.2987	0.9713	0.9841	0.149*	0.61 (3)
C2	1.2570 (17)	0.877 (3)	0.973 (2)	0.092 (10)	0.39 (3)
H2A	1.3158	0.8233	1.0015	0.138*	0.39 (3)
H2B	1.2917	0.9634	0.9722	0.138*	0.39 (3)
H2C	1.2532	0.8444	0.9236	0.138*	0.39 (3)
C3	1.0458 (6)	1.0852 (6)	0.8430 (3)	0.0711 (15)	
H3A	1.1294	1.1326	0.8520	0.085*	
H3B	1.0655	1.0203	0.8063	0.085*	
C4	0.9436 (7)	1.1744 (8)	0.8136 (4)	0.110 (2)	
H4A	0.9777	1.2132	0.7697	0.165*	
H4B	0.9248	1.2403	0.8490	0.165*	
H4C	0.8613	1.1280	0.8029	0.165*	
C5	0.9034 (6)	1.2082 (5)	1.0420 (3)	0.0578 (13)	
C6	0.7695 (5)	1.1334 (5)	1.0345 (3)	0.0540 (12)	
H6	0.6974	1.1949	1.0220	0.065*	
C7	0.7302 (6)	1.0675 (5)	1.1066 (3)	0.0630 (13)	
H7	0.7271	1.1347	1.1441	0.076*	
C8	0.8363 (6)	0.9696 (6)	1.1306 (3)	0.0813 (17)	
H8A	0.9244	1.0099	1.1322	0.122*	
H8B	0.8382	0.8992	1.0966	0.122*	
H8C	0.8135	0.9377	1.1783	0.122*	
C9	0.5892 (6)	1.0081 (7)	1.1028 (4)	0.0880 (18)	
H9A	0.5682	0.9678	1.1486	0.132*	
H9B	0.5869	0.9449	1.0646	0.132*	
H9C	0.5233	1.0742	1.0928	0.132*	
C10	0.6751 (4)	1.0202 (4)	0.9345 (3)	0.0522 (10)	
H10	0.5978	1.0684	0.9454	0.063*	
C11	0.6638 (5)	0.9281 (4)	0.8755 (2)	0.0491 (11)	
C12	0.7670 (5)	0.8381 (5)	0.8588 (2)	0.0529 (11)	
C13	0.7368 (6)	0.7468 (5)	0.8052 (3)	0.0656 (14)	
H13	0.8009	0.6840	0.7937	0.079*	
C14	0.6135 (7)	0.7484 (5)	0.7690 (3)	0.0684 (15)	
H14	0.5953	0.6873	0.7332	0.082*	
C15	0.5183 (6)	0.8391 (5)	0.7856 (2)	0.0594 (12)	
C16	0.5395 (5)	0.9272 (5)	0.8387 (3)	0.0561 (12)	
H16	0.4720	0.9865	0.8505	0.067*	

### Atomic displacement parameters (Å^2^)

**Table:**

	*U*^11^	*U*^22^	*U*^33^	*U*^12^	*U*^13^	*U*^23^
Sn1	0.05146 (17)	0.04937 (17)	0.05644 (19)	0.00307 (15)	−0.00404 (14)	0.00692 (14)
N1	0.050 (2)	0.0361 (19)	0.051 (2)	0.0014 (15)	−0.0056 (17)	−0.0018 (16)
O1	0.063 (2)	0.0596 (18)	0.075 (2)	−0.011 (2)	−0.0039 (19)	−0.0065 (17)
O2	0.093 (3)	0.052 (2)	0.082 (3)	−0.0179 (19)	−0.006 (2)	−0.0097 (19)
O3	0.0571 (19)	0.0482 (18)	0.072 (2)	0.0089 (15)	−0.0039 (17)	−0.0096 (17)
Br1	0.0958 (5)	0.1095 (6)	0.0913 (5)	−0.0262 (4)	−0.0366 (4)	−0.0077 (4)
C1	0.071 (4)	0.071 (4)	0.088 (4)	0.013 (3)	−0.013 (3)	0.021 (3)
C2'	0.063 (7)	0.096 (10)	0.139 (18)	−0.009 (6)	−0.044 (8)	0.042 (11)
C2	0.066 (10)	0.091 (17)	0.12 (2)	0.017 (9)	−0.017 (11)	0.034 (15)
C3	0.072 (3)	0.082 (4)	0.059 (3)	0.002 (3)	0.002 (3)	0.020 (3)
C4	0.087 (4)	0.127 (6)	0.116 (6)	0.013 (4)	0.000 (4)	0.055 (5)
C5	0.078 (3)	0.042 (3)	0.053 (3)	−0.010 (2)	−0.010 (3)	0.007 (2)
C6	0.061 (3)	0.047 (3)	0.053 (3)	0.003 (2)	−0.010 (2)	−0.011 (2)
C7	0.073 (3)	0.056 (3)	0.060 (3)	−0.006 (3)	0.000 (3)	−0.009 (3)
C8	0.112 (5)	0.073 (4)	0.059 (3)	−0.009 (3)	0.001 (3)	0.015 (3)
C9	0.083 (4)	0.093 (4)	0.089 (4)	−0.019 (4)	0.026 (3)	−0.010 (4)
C10	0.055 (2)	0.044 (2)	0.057 (3)	0.001 (2)	0.002 (2)	−0.005 (2)
C11	0.059 (3)	0.047 (3)	0.041 (3)	−0.005 (2)	−0.001 (2)	0.002 (2)
C12	0.064 (3)	0.048 (3)	0.047 (3)	−0.006 (2)	0.011 (2)	0.001 (2)
C13	0.080 (4)	0.059 (3)	0.059 (3)	−0.005 (3)	0.010 (3)	−0.013 (3)
C14	0.092 (4)	0.061 (3)	0.052 (3)	−0.017 (3)	0.008 (3)	−0.015 (3)
C15	0.065 (3)	0.066 (3)	0.047 (3)	−0.018 (3)	−0.007 (2)	−0.001 (2)
C16	0.061 (3)	0.052 (3)	0.055 (3)	−0.003 (2)	−0.007 (2)	0.001 (2)

### Geometric parameters (Å, °)

**Table:**

Sn1—O3	2.106 (3)	C4—H4B	0.9600
Sn1—C1	2.114 (5)	C4—H4C	0.9600
Sn1—C3	2.126 (5)	C5—C6	1.532 (7)
Sn1—O1	2.153 (3)	C6—C7	1.536 (8)
Sn1—N1	2.153 (4)	C6—H6	0.9800
N1—C10	1.296 (6)	C7—C9	1.515 (7)
N1—C6	1.470 (6)	C7—C8	1.520 (8)
O1—C5	1.287 (6)	C7—H7	0.9800
O2—C5	1.221 (6)	C8—H8A	0.9600
O3—C12	1.310 (5)	C8—H8B	0.9600
Br1—C15	1.905 (5)	C8—H8C	0.9600
C1—C2	1.454 (19)	C9—H9A	0.9600
C1—C2'	1.504 (14)	C9—H9B	0.9600
C1—H1A	0.9700	C9—H9C	0.9600
C1—H1B	0.9700	C10—C11	1.446 (6)
C2'—H2D	0.9600	C10—H10	0.9300
C2'—H2E	0.9600	C11—C16	1.393 (6)
C2'—H2F	0.9600	C11—C12	1.411 (6)
C2—H2A	0.9600	C12—C13	1.396 (7)
C2—H2B	0.9600	C13—C14	1.379 (8)
C2—H2C	0.9600	C13—H13	0.9300
C3—C4	1.467 (8)	C14—C15	1.360 (7)
C3—H3A	0.9700	C14—H14	0.9300
C3—H3B	0.9700	C15—C16	1.350 (7)
C4—H4A	0.9600	C16—H16	0.9300
			
O3—Sn1—C1	95.3 (2)	O2—C5—O1	126.0 (5)
O3—Sn1—C3	97.16 (19)	O2—C5—C6	118.0 (5)
C1—Sn1—C3	123.3 (2)	O1—C5—C6	116.0 (4)
O3—Sn1—O1	156.21 (13)	N1—C6—C5	108.7 (4)
C1—Sn1—O1	95.7 (2)	N1—C6—C7	112.5 (4)
C3—Sn1—O1	94.3 (2)	C5—C6—C7	111.4 (4)
O3—Sn1—N1	82.21 (13)	N1—C6—H6	108.0
C1—Sn1—N1	124.4 (2)	C5—C6—H6	108.0
C3—Sn1—N1	112.03 (18)	C7—C6—H6	108.0
O1—Sn1—N1	74.16 (14)	C9—C7—C8	111.5 (5)
C10—N1—C6	117.3 (4)	C9—C7—C6	111.8 (5)
C10—N1—Sn1	124.2 (3)	C8—C7—C6	112.0 (5)
C6—N1—Sn1	116.7 (3)	C9—C7—H7	107.1
C5—O1—Sn1	121.1 (3)	C8—C7—H7	107.1
C12—O3—Sn1	126.6 (3)	C6—C7—H7	107.1
C2—C1—C2'	48.3 (10)	C7—C8—H8A	109.5
C2—C1—Sn1	110.8 (8)	C7—C8—H8B	109.5
C2'—C1—Sn1	114.4 (6)	H8A—C8—H8B	109.5
C2—C1—H1A	140.2	C7—C8—H8C	109.5
C2'—C1—H1A	108.7	H8A—C8—H8C	109.5
Sn1—C1—H1A	108.7	H8B—C8—H8C	109.5
C2—C1—H1B	64.6	C7—C9—H9A	109.5
C2'—C1—H1B	108.7	C7—C9—H9B	109.5
Sn1—C1—H1B	108.7	H9A—C9—H9B	109.5
H1A—C1—H1B	107.6	C7—C9—H9C	109.5
C1—C2'—H2D	109.5	H9A—C9—H9C	109.5
C1—C2'—H2E	109.5	H9B—C9—H9C	109.5
H2D—C2'—H2E	109.5	N1—C10—C11	126.1 (4)
C1—C2'—H2F	109.5	N1—C10—H10	116.9
H2D—C2'—H2F	109.5	C11—C10—H10	116.9
H2E—C2'—H2F	109.5	C16—C11—C12	121.2 (4)
C1—C2—H2A	109.5	C16—C11—C10	115.6 (4)
C1—C2—H2B	109.5	C12—C11—C10	123.0 (4)
H2A—C2—H2B	109.5	O3—C12—C13	119.8 (5)
C1—C2—H2C	109.5	O3—C12—C11	123.4 (4)
H2A—C2—H2C	109.5	C13—C12—C11	116.7 (5)
H2B—C2—H2C	109.5	C14—C13—C12	121.0 (5)
C4—C3—Sn1	114.4 (4)	C14—C13—H13	119.5
C4—C3—H3A	108.7	C12—C13—H13	119.5
Sn1—C3—H3A	108.7	C15—C14—C13	120.2 (5)
C4—C3—H3B	108.7	C15—C14—H14	119.9
Sn1—C3—H3B	108.7	C13—C14—H14	119.9
H3A—C3—H3B	107.6	C16—C15—C14	121.6 (5)
C3—C4—H4A	109.5	C16—C15—Br1	119.4 (4)
C3—C4—H4B	109.5	C14—C15—Br1	119.0 (4)
H4A—C4—H4B	109.5	C15—C16—C11	119.2 (5)
C3—C4—H4C	109.5	C15—C16—H16	120.4
H4A—C4—H4C	109.5	C11—C16—H16	120.4
H4B—C4—H4C	109.5		
			
O3—Sn1—N1—C10	−34.1 (4)	Sn1—N1—C6—C5	20.5 (5)
C1—Sn1—N1—C10	−125.3 (4)	C10—N1—C6—C7	91.3 (5)
C3—Sn1—N1—C10	60.5 (4)	Sn1—N1—C6—C7	−103.3 (4)
O1—Sn1—N1—C10	148.7 (4)	O2—C5—C6—N1	167.5 (4)
O3—Sn1—N1—C6	161.6 (3)	O1—C5—C6—N1	−14.2 (6)
C1—Sn1—N1—C6	70.5 (4)	O2—C5—C6—C7	−67.9 (6)
C3—Sn1—N1—C6	−103.8 (3)	O1—C5—C6—C7	110.3 (5)
O1—Sn1—N1—C6	−15.6 (3)	N1—C6—C7—C9	−65.2 (6)
O3—Sn1—O1—C5	0.6 (6)	C5—C6—C7—C9	172.5 (5)
C1—Sn1—O1—C5	−116.7 (4)	N1—C6—C7—C8	60.8 (6)
C3—Sn1—O1—C5	119.2 (4)	C5—C6—C7—C8	−61.6 (6)
N1—Sn1—O1—C5	7.5 (3)	C6—N1—C10—C11	−176.0 (4)
C1—Sn1—O3—C12	163.1 (4)	Sn1—N1—C10—C11	19.9 (6)
C3—Sn1—O3—C12	−72.3 (4)	N1—C10—C11—C16	−176.7 (5)
O1—Sn1—O3—C12	45.8 (6)	N1—C10—C11—C12	7.8 (7)
N1—Sn1—O3—C12	39.0 (4)	Sn1—O3—C12—C13	154.0 (4)
O3—Sn1—C1—C2	104.8 (18)	Sn1—O3—C12—C11	−28.1 (6)
C3—Sn1—C1—C2	2.6 (19)	C16—C11—C12—O3	−179.4 (4)
O1—Sn1—C1—C2	−96.3 (18)	C10—C11—C12—O3	−4.1 (7)
N1—Sn1—C1—C2	−171.0 (18)	C16—C11—C12—C13	−1.5 (7)
O3—Sn1—C1—C2'	157.3 (13)	C10—C11—C12—C13	173.8 (4)
C3—Sn1—C1—C2'	55.1 (14)	O3—C12—C13—C14	−179.9 (5)
O1—Sn1—C1—C2'	−43.9 (14)	C11—C12—C13—C14	2.1 (7)
N1—Sn1—C1—C2'	−118.5 (13)	C12—C13—C14—C15	−0.5 (8)
O3—Sn1—C3—C4	87.1 (5)	C13—C14—C15—C16	−1.9 (8)
C1—Sn1—C3—C4	−171.7 (5)	C13—C14—C15—Br1	178.8 (4)
O1—Sn1—C3—C4	−72.0 (5)	C14—C15—C16—C11	2.4 (8)
N1—Sn1—C3—C4	2.7 (6)	Br1—C15—C16—C11	−178.2 (4)
Sn1—O1—C5—O2	179.8 (4)	C12—C11—C16—C15	−0.7 (7)
Sn1—O1—C5—C6	1.7 (5)	C10—C11—C16—C15	−176.3 (4)
C10—N1—C6—C5	−144.9 (4)		
